# Effects of Surgeon Handedness on the Outcomes of Unicompartmental Knee Arthroplasty: A Single Center's Experience

**DOI:** 10.1111/os.13549

**Published:** 2022-10-25

**Authors:** Zheng Cao, Yubo Liu, Minzhi Yang, Zhuo Zhang, Xiangpeng Kong, Wei Chai

**Affiliations:** ^1^ Senior Department of Orthopedics The Fourth Medical Center of PLA General Hospital Beijing China; ^2^ Medical School of Nankai University Tianjin China; ^3^ National Clinical Research Center for Orthopedics, Sports Medical and Rehabilitation Beijing China

**Keywords:** Clinical outcomes, Prosthesis orientation, Surgeon handedness, Unicompartmental knee arthroplasty

## Abstract

**Objective:**

Surgeon handedness has been widely discussed in operative surgery, and could cause clinical discrepancy. However, few studies have reported the effect of handedness on unicompartmental knee arthroplasty (UKA). Based on our clinical observation and case analysis, we aimed to find out the effects of surgeon handedness on UKA.

**Methods:**

We retrospectively studied 94 UKA procedures performed by one right‐handed surgeon from January 2017 to December 2018 at a single medical center. The cases were divided into two groups by operation side (49 L‐UKAs and 45 R‐UKAs). Preoperative demographic data were collected. Imaging parameters (femorotibial and hip‐knee‐ankle angles and tibial‐plateau retroversion) and joint function scores (Knee Society Score [KSS] and Oxford Knee Score [OKS]) were recorded. Patients were followed up regularly and Forgotten Joint Score (FJS) was calculated at the last follow‐up. All data were compared between the two groups with independent‐samples *t*‐test, and paired *t*‐test was used for intragroup comparisons.

**Results:**

The average follow‐up was 26.7 ± 3.2 months. The average patient age was 63.5 ± 9.0 years and the average body mass index was 26.89 ± 3.43 kg/m^2^. There was no significant group difference in any preoperative characteristic. Both the KSS and OKS improved significantly after surgery (*p* < 0.05). No significant group difference was found between the KSS or OKS at any follow‐up visit. The varus or valgus of tibial component was 3.57 ± 1.42° on the left side and 3.19 ± 1.56° on the right side (*p* = 0.45). The varus or valgus of femoral component was 7.81 ± 2.43° in patients undergoing L‐UKA and 7.05 ± 2.90° in those undergoing R‐UKA (*p* = 0.04). No statistical differences were found in outliers of component orientation on both sides. The femorotibial and hip‐knee‐ankle angles improved significantly in both groups, and there was no significant group difference in either lower limb alignment or tibial‐plateau retroversion. The complication rate was 8.16% (4/49) in the L‐UKA group and 6.67% (3/45) in the R‐UKA group. There was no correlation between prosthesis orientation and early joint function score.

**Conclusions:**

Surgeon handedness may cause a worse prosthetic orientation on femoral side during surgeon's non‐dominant UKA, and surgeons should be cautious of bone resection and prosthesis implantation. However, radiographic difference did not bring variations on short‐term clinical outcomes or lower limb alignment.

## Graphical abstract

We aimed to evaluate the effects of surgeon handedness on conventional unicompartmental knee arthroplasty.

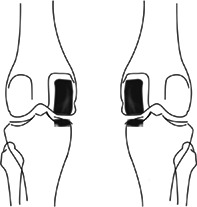

## Introduction

Anteromedial knee osteoarthritis (AMOA) is always performed as cartilage damage in medial condylar.^1^, ^2^ And unicompartmental knee arthroplasty (UKA) can be highly effective for AMOA patients if conservative treatments (muscle exercises and non‐steroidal anti‐inflammatory drugs [NSAIDs]) and articular injections are ineffective.^3^ With strict criteria, UKA provides better joint function and range of motion at early follow‐up than total knee arthroplasty (TKA).^4^, ^5^ However, the survival rate of UKA is still controversial,^6^, ^7^, ^8^, ^9^ and suited prosthesis orientation is the precondition of satisfied survival rate. Although digital‐assisted technology helps prosthesis implantation, most UKAs are performed manually in China due to expensive cost and uncertain financial effect.

In our single institution's clinical observation, the radiographical outcomes of left UKAs (L‐UKAs) performed by a right‐handed surgeon seemed to be worse than those of right UKAs (R‐UKAs). Thus, we were determined to explore effects of handedness on conventional UKA. Handedness refers to the tendency to use one hand more than the other. Surgeon handedness has been widely discussed in the literature on laparoscopic abdominal surgery^10^ but has seldom been mentioned in orthopaedic reports. Mehta *et al*. found better clinical outcomes including extension and the Knee Society Score in TKA with surgeons' dominant hand.^11^ Song *et al*. concluded that the placement of cup performed by dominant hands of surgeons was more accurate than that performed by non‐dominant sides.^12^ And Pennington *et al*. observed higher inclination angles when operating on the dominant side.^13^ All of the studies mentioned above reported the effects of handedness on joint replacement surgery.

However, our observations about conventional UKA and surgeon handedness lacked the support of statistical analysis, and to the best of our knowledge, there have been few reports of the effect of surgeon handedness on UKA outcomes. Therefore, we aimed to find out the effects of surgeon handedness on UKA; to assess whether patients who underwent right UKA (R‐UKA) had better radiographic and functional outcomes than patients who underwent left UKA (L‐UKA) when the surgeon was right‐handed; to analyze the relationship between prosthesis orientation and joint function. We hypothesized that, for a right‐handed surgeon, R‐UKAs would provide better radiographic and functional outcomes than L‐UKAs on both the femoral and tibial sides. We also hypothesized that the clinical and radiographic outcomes would be correlated.

## Materials and Methods

### Design, Participants, and Interventions

In total, 94 UKA procedures performed in the Orthopaedic Department of the Chinese People's Liberation Army General Hospital from January 2017 to December 2018 were retrospectively studied. The trial was approved by the Clinical Trial Ethics Committee of our hospital (Institutional Review Board approval number: 2019‐042‐7).

The study's inclusion criteria were as follows: diagnosed as AMOA or osteonecrosis; aged 50–75 years; a suitable UKA candidate (as judged by the surgeon); complete preoperative data; and at least 1 year of follow‐up data. The exclusion criteria were as follows: hip or knee surgery of the ipsilateral lower extremity in the past 12 months; >10° of fixed flexion contracture or varus deformity of the operated knee; infection; inflammatory arthropathy; extensive ligament relaxation; insufficient bone mass because of disease, or implant stability.

The study was approved by the clinical ethics committee of the Chinese PLA General Hospital and all patients gave written informed consent. All procedures were performed by one experienced right‐handed surgeon (CW). All cases were divided into two groups according to the operating side (49/94 left; 45/94 right). All patients were admitted to the Orthopaedic Department of the Chinese PLA General Hospital after outpatient evaluation by CW. Before surgery, anteroposterior and lateral radiographs of the knee joints and lower limbs were taken. MRI was performed if cartilage‐bearing structures and cruciate ligament status were unclear. All preoperative physical examinations were performed by CW, who carefully evaluated all symptoms (particularly the pain locations). All 94 UKA procedures were successfully accomplished.

### Surgical Technique

We followed a standard surgical procedure. Each patient was placed in the half‐lithotomy position and general anesthesia was induced. A tourniquet was applied. The surgeon created a small incision *via* a parapatellar approach. The integrity of the anterior and posterior cruciate ligaments was confirmed after removal of the medial meniscus. Bone cutting involved removal of femoral, tibial, and intercondylar fossa osteophytes. Gap balancing and impingement were tested. The range of motion was re‐checked after implantation of all components and consolidation of the cement. For all procedures, a well‐established fixed‐bearing prosthesis was used (Oxford Unicompartmental Knee System; Zimmer Biomet, Warsaw, IN, USA). The incision was sutured layer‐by‐layer and surgery was then concluded.

### Perioperative Management

All patients were routinely given intravenous low‐molecular heparin sodium during the first 1–2 days after surgery, and aspirin for 1 month to prevent lower‐limb thrombosis. All patients received routine intravenous antibiotics (ceftriaxone sodium or levofloxacin) for 1–3 days after surgery, and oral antibiotics for 1 week. Oxycodone and NSAIDs were prescribed in the early postoperative period, and NSAID use continued to 3 months after discharge. Anteroposterior and lateral radiographs of the knee joints and lower limbs were taken on the first day after surgery. Arteriovenous ultrasonography of the lower extremities was performed if necessary.

### Postoperative Rehabilitation

The rehabilitation protocol was standardized. Patients were allowed to walk with full weight‐bearing (using mobility aids) on day 1 after surgery. The straight leg‐raising exercise was recommended. Antibiotics and anticoagulants were administered to protect against infection and thrombosis. All patients were followed‐up at 6 weeks, 3 months, and 1 year after surgery. During each follow‐up, the joint function score was obtained by the surgical team, and standing anteroposterior and lateral X‐rays of the knee joint were taken.

### Clinical Outcomes

#### Knee Society Score

We used the KSS to assess clinical outcomes. The KSS evaluates objective and subjective clinical and functional knee parameters. The maximum score (reflecting the best possible outcome) is 200. Total scores ≤70 were considered poor, while scores of 71–80 were fair, scores of 81–90 were good, and scores of 91–100 were excellent.

#### Oxford Knee Score

The Oxford Knee Score (OKS) is more commonly used in UKA studies than the KSS because it reflects subjective knee joint status before and after surgery. The OKS (including the Chinese version) has been validated.^14^, ^15^ The 12‐part OKS assesses the pain associated with five daily activities and seven functions. Each part is scored from 1 (no pain) to 5 (severe pain) and the final score is derived by summing the 12 scores.

#### Forgotten Joint Score

The Forgotten Joint Score (FJS) is a recently introduced patient‐reported outcome measure. The FJS is used to assess the ability of joint replacement patients to complete various activities. The total score is 100; higher scores indicate poorer performance. The FJS has been validated, and scores thereon correlate with earlier joint function scores.^16^

### Radiographic Measurements

#### Lower‐Limb Alignment Measurements

Full length X‐ray of lower limb was required to measure hip‐knee‐ankle angle (HKA) and femorotibial angle (FTA). HKA was defined as the angle of line from hip center to knee center and line from knee center to ankle center. And FTA was defined as the angle of femoral long axis and tibia long axis. Both HKA and FTA could reflex lower‐limb alignment. And the measurements were conducted before surgery, 3 months after surgery, and annually thereafter.

#### Component Orientation Measurements

Standing anteroposterior and lateral knee X‐rays were acquired to measure component orientations using the method of Shakespeare.^17^ The coronal orientation of the femoral and tibial components was given by the angle between the two components and anatomical axis of the femur and tibia, respectively. The tibia plateau retroversion was the angle between the cortical tangent on the posterior side of the tibial component and longitudinal axis (vertical line) of the tibial component. As recommended by the developers of the Oxford Unicompartmental Knee Arthroplasty system,^18^ the tibial and femoral components' varus or valgus were recorded separately. And 0° was defined as perfect orientation of component on coronal plane. The radiographic outcomes of each knee were evaluated by two independent clinicians, and average values were calculated.

### Intraoperative Findings and Complications

We recorded operation time (from incision to closure) and blood loss (using the method of Gross^19^). Intra‐ and postoperative complications were noted. Intraoperative complications included periprosthetic fractures, neurovascular injury or infection, and joint hematoma. Postoperative complications included ectopic ossification, component loosening, dislocation, and persistent pain. The complication rates were calculated separately for the two groups.

### Statistical Analysis

SPSS software (version 22.0; IBM Corp., Armonk, NY, USA) was used for all analyses. Data are expressed as means ± standard deviations. The independent‐samples *t*‐test was used for intergroup comparisons, and the paired *t*‐test for intragroup comparisons, both before and after surgery. Fisher's exact test was used to compare counts. Intraclass correlation coefficients (ICC)s was calculated to determine measurement reliability (0.81–1.00, near‐perfect; 0.61–0.80, strong; 0.41–0.60, moderate; 0.21–0.40, fair; 0–0.20, poor). Pearson correlation analysis was used to assess associations between component locations and functional scores. The level of significance was set to *p* < 0.05.

## Results

### General Results

The L‐UKA and R‐UKA groups included 49 and 45 patients, respectively. The mean age of the patients was 63.50 ± 9.0 years, and the mean body mass index (BMI) was 26.89 ± 3.43 kg/m^2^. There was no significant difference in age, gender, BMI, preoperative KSS, or preoperative OKS between the two groups (Table 1).

**Table 1:** Preoperative demographics of left‐handed group and right‐handed group

Baseline data	Totality (*n* = 94)	Left‐handed (*n* = 49)	Right‐handed (*n* = 45)	*p*	T/X^2^
Age (yrs)	63.5(9.0)	64.2(8.4)	62.7(9.7)	0.201	0.834
BMI (kg/m^2^)	26.89(3.43)	26.41(3.36)	27.41(3.48)	0.972	−1.414
Gender (male/female)	30/64	15/34	15/30	0.777	0,080
Follow‐up period (months)	26.7(3.2)	26.6(3.3)	26.7(3.1)	0.752	−0.933
Preoperative KSS	80.79(7.40)	81.66(7.79)	79.84(6.92)	0.633	1.193
Preoperative OKS	32.46(3.96)	32.90(3.44)	31.98(4.45)	0.111	1.436

*Note*: Data format: Mean (standard deviation).

Abbreviation: BMI, Body Mass Index; KSS, Knee Society Score; OKS, Oxford Knee Score.

### Intraoperative Data

There was no group difference in blood loss or operation time (P>0.05). The complication rates were 8.16% (4/49) in L‐UKA group and 6.67% (3/45) in R‐UKA group, respectively, and they were not significantly different (Table 4).

### Radiographical Outcomes

All patients were followed up at 3 and 6 months, and annually thereafter. The average follow‐up period was 26.7 months. The radiographic images of the last follow‐up in outpatient were adopted to measure.

#### Lower‐Limb Alignment Measurements

The postoperative HKA was 5.31 ± 2.90° in L‐UKA group and 4.82 ± 3.01° in R‐UKA group. The postoperative FTA was −3.29 ± 4.63° in L‐UKA group and − 2.88 ± 4.42° in R‐UKA group. Both HKA and FTA improved in two groups, and no significant group difference was apparent (P>0.05).

#### Prosthesis Orientation Measurements

The optimal tibia‐plateau retroversion angle was 7°, and both groups almost achieved this angle (6.74 ± 3.33° *vs* 6.95 ± 3.50°, *p* = 0.767). The varus or valgus of femoral component in R‐UKA group were significantly better than those in L‐UKA group (7.81 ± 2.43° *vs*. 7.05 ± 2.90°, P<0.05). And the varus or valgus of tibia component did not differ significantly between the groups (3.57 ± 1.42° *vs*. 3.19 ± 1.56°, *p* = 0.454). There were more coronal plane femoral and tibial prosthesis angle outliers in the L‐UKA than R‐UKA group, but the difference was not statistically significant (Table 2).

**Table 2:** Changes of lower limb ligament and results of postoperative prosthesis orientation

Imaging parameters	Left‐handed (*n* = 49)	Right‐handed (*n* = 45)	*p*	T/X^2^
Pre‐op HKA ankle	5.80(2.97)	6.17(2.80)	0.615	−0.612
Post‐op HKA ankle	5.31(2.90)*	4.82(3.01)*	0.890	1.135
Pre‐op femorotibial ankle	−0.47(4.11)	0.10(3.87)	0.617	−0.687
Post‐op femorotibial ankle	−3.29(4.63)**	−2.88(4.42)**	0.325	−0.441
Pre‐op tibia plateau retroversion	4.28(2.66)	4.52(2.45)	0.863	−1.032
Post‐op tibia component retroversion	6.74(3.33)*	6.95(3.50)*	0.767	−0.976
Femur component varus/valgus	7.81(2.43)	7.05(2.90)	0.037	1.381
Femur side outliers	6/49(12.24%)	2/45(4.44%)	0.176	1.833
Tibia component varus/valgus	3.57(1.42)	3.19(1.56)	0.454	0.606
Tibia side outliers	2/49(4.08%)	1/45(2.22%)	0.608	0.262

*Note*: Data format: Mean (standard deviation) Intra‐group paired *t* test.

* *p* < 0.05.

** *p* < 0.001.

### Clinical Outcomes

The preoperative KSS was 81.66 ± 7.79 in L‐UKA group and 79.84 ± 6.92 in R‐UKA group. The preoperative OKS was 32.90 ± 3.44 in L‐UKA group and 31.98 ± 4.45 in R‐UKA group. We found no significant group difference in the KSS or OKS scores at any follow‐up time point, and FJS was also similar between two groups (Table 3). Table 5 shows the most recent joint function scores according to component orientation; there were no significant correlations.

**Table 3:** Comparison of postoperative joint function score between two groups

Joint function score	Left‐handed (*n* = 49)	Right‐handed (*n* = 45)	*p*
KSS (3 months postoperatively)	165.74(13.43)	171.43(14.22)	0.131
KSS (1 year postoperatively)	181.23(8.11)	179.97(7.34)	0.793
KSS (latest follow‐up)	183.76(9.27)	180.68(10.30)	0.552
OKS (3 months postoperatively)	18.27(5.45)	20.73(6.13)	0.870
OKS (1 year postoperatively)	13.63(7.50)	14.75(6.97)	0.499
OKS (latest follow‐up)	12.39(6.68)	13.02(7.71)	0.737
FJS (1 year postoperatively)	88.35(8.43)	90.20(6.73)	0.859
FJS (latest follow‐up)	92.42(9.09)	93.97(8.33)	0.901

*Note*: Data format: Mean (standard deviation).

Abbreviation: KSS, Knee Society Score; OKS, Oxford Knee Score; FJS, Forgotten Joint Score.

**Table 4:** Perioperative information and complications of each group

	Totality (*n* = 94)	Left‐handed (*n* = 49)	Right‐handed (n = 45)	*p*	T/X2
Operation time (minutes)	55.04(6.44)	55.47(5.86)	54.58(7.05)	0.505	0.668
Blood loss (ml)	102.50(11.58)	103.77(9.82)	101.11(13.21)	0.267	1.116
Perioperative complications	1/94	0/49	1/45	0.294	1.101
Postoperative complications	6/94	4/49	2/45	0.461	0.543

**Table 5:** Correlation between postoperative radiographic results and joint function scores

Parameters	Latest OKS	Latest KSS	Latest FJS
Femur component varus/valgus	*r* = −0.035	*r* = 0.089	*r* = −0.018
	*p* = 0.738	*p* = 0.395	*p* = 0.862
Tibia component varus/valgus	*r* = 0.146	*r* = 0.064	*r* = −0.117
	*p* = 0.161	*p* = 0.541	*p* = 0.262
tibia component retroversion	*r* = −0.083	*r* = 0.044	*r* = 0.141
	*p* = 0.426	*p* = 0.676	*p* = 0.176

Abbreviation: KSS, Knee Society Score; OKS, Oxford Knee Score; FJS, Forgotten Joint Score.

### Complications

**Fig. 1:**
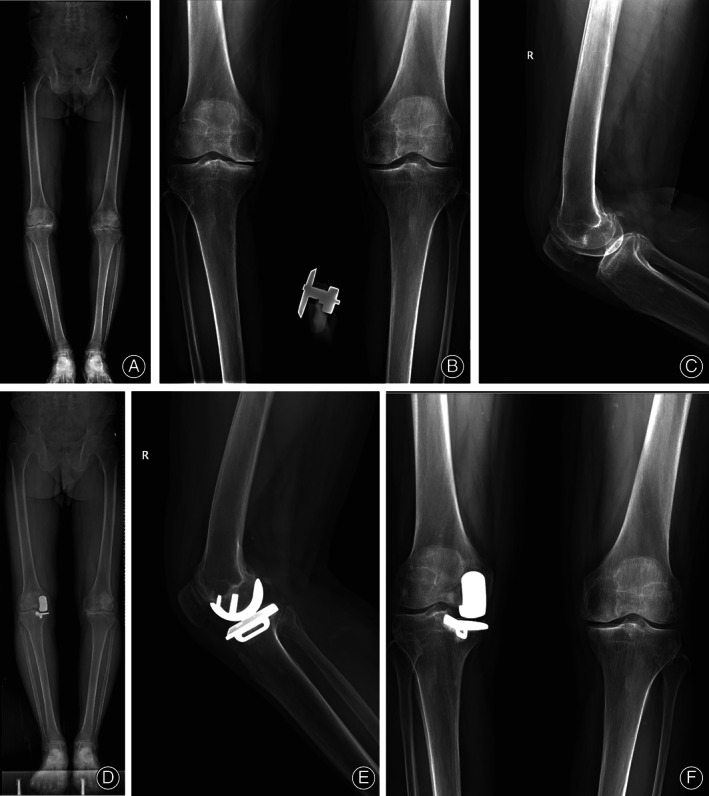
Male, 65 years old, diagnosed as AMOA and underwent UKA, showed a perfect radiographic result 1 year after surgery. (A) Lower limb length of X‐ray preoperatively. (B) Anteroposterior X‐ray preoperatively. (C) Lateral X‐ray preoperatively. (D) Lower limb length of X‐ray postoperatively. (E) Anteroposterior X‐ray postoperatively. (F) Lateral X‐ray postoperatively.

One L‐UKA patient reported joint instability 6 weeks after surgery; we replaced the original liner with a larger one. Three patients reported persistent (>3 months) pain. In R‐UKA group, two patients reported persistent pain and a joint hematoma was found in one patient. No R‐UKA case required revision surgery. Joint hematoma was adopted for conservative treatment and the patient was demanded to postpone function exercise. For patients with persistent pain, we provided NSAIDs for symptomatic treatment and all five patients claimed an improvement. Figure 1 shows a typical case in the research.

## Discussion

Our research concluded that the radiographic outcomes of R‐UKAs were significantly better than those of L‐UKAs on femoral side, but with no significant differences on tibia side for a right‐handed surgeon. More complications were found in L‐UKA group, although the difference was not statistically significant. Also, there was no relationship between postoperative joint function score and radiographic results.

### Surgeon Handedness

Some studies evaluated the effects of the handedness of orthopaedic surgeons. Melony *et al*. found that patients with left femoral neck fractures operated on by right‐handed surgeons had more complications than those with right femoral neck fractures.^20^ Mehta *et al*. suggested that surgeon handedness may influence TKA prognosis.^11^ Both Song and Pennington *et al*. found that prosthesis position may reflect the handedness of the surgeon performing TKA.^12^, ^13^ When performing UKA, a small incision and good surgical field are essential; joint surgeons may require more time to perform UKA than TKA. As prosthesis orientation greatly affects long‐term survival, we focused on the radiographic data for the two operation sides. To the best of our knowledge, few studies have explored whether surgeon handedness affects the outcomes of UKA. Our sample was relatively large, and all peri‐ and postoperative procedures were standardized; thus, our conclusions are credible.

### Radiographic Results and Clinical Outcomes

The radiographic outcomes of out L‐UKA patients were poorer than those of the R‐UKA patients. We suspect that, during left joint surgery, the left hand completed more steps than the right hand. Stability and accuracy are always poorer when using the left hand. Furthermore, even when standard bone‐cutting tools are used, the prosthesis orientation may be suboptimal on both the femoral and tibial sides. The poorer radiographic results on the tibial side seen in this study suggest that care is needed when operating on that side. Operation time and blood loss were reduced when the dominant hand was used (P>0.05). We suspect that the UKA procedure was shorter on the right side, thus decreasing blood loss. However, further research is needed to confirm this.

We found no significant relationship between prosthesis orientation and joint function score, consistent with previous results.^21^ The UKA outcomes were not associated with component orientation. Kayani *et al*. reported excellent outcomes at the mid‐to‐long‐term follow‐up.^5^ It remains unclear whether prosthesis position affects long‐term outcomes. The complication rate in our patients undergoing L‐UKA was higher, although not statistically significantly, than that of the R‐UKA patients. A study with a larger sample size and more surgeons is needed to further explore this.

### Limitations

This study had some limitations. Firstly, the follow‐up period of our study was relatively short, and long‐term follow‐up was expected to compare differences of survival rate or joint function. Even so, our study was one of the earliest to focus on UKA's surgeon handedness. Secondly, the sample size of our study was relatively small, although our sample was the largest of any study of the effects of surgeon handedness on the outcomes of patients undergoing UKA, it was nevertheless still modest. Thirdly, although all of the patients were treated identically, confounding factors remain a possibility. A larger sample and multivariate regression analysis would provide more valid data.

### Conclusion

Surgeon handedness may affect prosthetic orientation on femoral side during conventional UKA. However, there was no group difference in short‐term clinical outcomes, complication rate, or lower limb alignment. No relationship was founded between short‐term clinical outcomes and prosthesis orientation. Regardless, we advised that joint surgeons should be particularly careful when operating on their non‐dominant side to avoid poor prosthesis orientation.

## Author Contributions

All authors have made substantial contributions to: (1) the conception and design of the study, (2) drafting the article or revising it critically for important intellectual content, (3) final approval of the version to be submitted. Zheng Cao and Yubo Liu: performed the experiments and wrote the manuscript. Minzhi Yang and Zhuo Zhang revised the manuscript and helped perform the analysis with constructive discussions. Wei Chai and Xiangpeng Kong: primarily responsible for oversight of the research project, including the study design, data analysis, manuscript preparation, and approval. Zheng Cao and Yubo Liu contributed equally to this work. Wei Chai and Xiangpeng Kong contributed equally to this work.

## Conflict of Interest

Each author certifies that he or she has no commercial associations (e.g. consultancies, stock ownership, equity interest, patent/licensing arrangements, etc.) that might pose a conflict of interest in connection with the submitted article.

## Ethics Statement

All ICMJE Conflict of Interest Forms for authors, clinical orthopaedists, related research editors, and board members are on file with the publication and can be viewed on request. All authors are in agreement with the manuscript. Each author certifies that his or her institution approved the human protocol for this investigation, that all investigations were conducted in conformity with ethical principles of research, and that informed consent for participation in the study was obtained.
